# Analysis of Self-Adhesive Resin Cement Microshear Bond Strength on Leucite-Reinforced Glass-Ceramic with/without Pure Silane Primer or Universal Adhesive Surface Treatment

**DOI:** 10.1155/2015/361893

**Published:** 2015-10-18

**Authors:** Yoon Lee, Jae-Hoon Kim, Jung-Soo Woo, Young-Ah Yi, Ji-Yun Hwang, Deog-Gyu Seo

**Affiliations:** ^1^Department of Conservative Dentistry, Wonju Severance Christian Hospital, Wonju College of Medicine, Yonsei University, Wonju 220-701, Republic of Korea; ^2^Department of Conservative Dentistry and Dental Research Institute, School of Dentistry, Seoul National University, Seoul 110-749, Republic of Korea; ^3^Dental Research Institute and School of Dentistry, Seoul National University, Seoul 110-749, Republic of Korea; ^4^Department of Dentistry, Inje University Seoul Paik Hospital, Seoul 100-032, Republic of Korea; ^5^Nutrition Education Major, Graduate School of Education, Sangmyung University, Seoul 110-743, Republic of Korea

## Abstract

*Objective*. To evaluate the microshear bond strength (*μ*SBS) of self-adhesive resin (SA) cement on leucite-reinforced glass-ceramic using silane or universal adhesive. *Materials and Methods*. Ceramic blocks were etched with 9.5% hydrofluoric acid and divided into three groups (*n* = 16): (1) negative control (NC) without treatment; (2) Single Bond Universal (SBU); (3) RelyX Ceramic Primer as positive control (PC). RelyX Unicem resin cement was light-cured, and *μ*SBS was evaluated with/without thermocycling. The *μ*SBS was analyzed using one-way analysis of variance. The fractured surfaces were examined using stereomicroscopy and scanning electron microscopy (SEM). *Results*. Without thermocycling, *μ*SBS was highest for PC (30.50 MPa ± 3.40), followed by SBU (27.33 MPa ± 2.81) and NC (20.18 MPa ± 2.01) (*P* < 0.05). Thermocycling significantly reduced *μ*SBS in SBU (22.49 MPa ± 4.11) (*P* < 0.05), but not in NC (20.68 MPa ± 4.60) and PC (28.77 MPa ± 3.52) (*P* > 0.05). PC and NC predominantly fractured by cohesive failure within the ceramic and mixed failure, respectively. *Conclusion*. SBU treatment improves *μ*SBS between SA cement and glass ceramics, but to a lower value than PC, and the improvement is eradicated by thermocycling. NC exhibited the lowest *μ*SBS, which remained unchanged after thermocycling.

## 1. Introduction

Because of its simplified cementation procedure, self-adhesive resin cement (SA cement) is becoming more widely used in dentistry. Specifically, it has been claimed that no additional surface treatment of the tooth surface is required when this new type of resin cement is applied [1, 2]. Therefore, this approach is time-saving and convenient for both the dentist and patient when compared with the conventional cementation procedure, which is time-consuming and technique-sensitive and which requires various products for use in multiple steps [3].

One of the most important properties of dental cement is high, durable bond strength, which is required for successful restorative treatment [3]. In previous studies, SA cements have exhibited clinically acceptable bond strength to both zirconia [4, 5] and metal restorations [6]. However, recent improvements in computer-aided design/computer-aided manufacturing (CAD-CAM) technology and patients' demand for esthetic treatment have led to increased use of ceramic restorations [7–9]. Hence, clinicians frequently use SA cements to bond ceramic restorations.

Silica-based ceramics have highly esthetic properties and have exhibited high bond strength to resin cement. In application, the surfaces of these ceramics are etched with 9.5% hydrofluoric acid to yield a micromechanically retentive surface [10, 11]. Then, for chemical bonding, silane is applied so that covalent and hydrogen bonds are formed [12–14]. This is followed by application of an adhesive [15]. Thus, manufacturers recommend silane pretreatment of silica-based ceramics before SA cements are used [16]. However, universal adhesives have recently been introduced for use in simple and convenient bonding procedures, and manufacturers claim they may be used on silica-based ceramics because they contain silane [16].

Based on the above findings, clinicians often use universal adhesives for silane application prior to application of SA cements. In contrast, some clinicians even assume that SA cements can be applied without any additional treatment of the ceramic surface. Thus, they apply SA cement directly onto an untreated ceramic surface, as in metal and zirconia restoration cementation procedures.

Scientific data regarding the effectiveness of silica-based ceramic surface treatment before cementation using SA cement is not widely available. Therefore, the purpose of this study is to evaluate the microshear bond strength (*μ*SBS) of self-adhesive resin cement on leucite-reinforced glass-ceramic both with and without surface treatment, using pure silane containing primer and universal adhesive before and after thermocycling. The null hypothesis is that no differences in the *μ*SBS between self-adhesive resin cement and leucite-reinforced glass-ceramic exist, regardless of whether pure-saline-containing primer or universal adhesive surface is used.

## 2. Materials and Methods

### 2.1. Specimen Preparation

**Figure 1:**
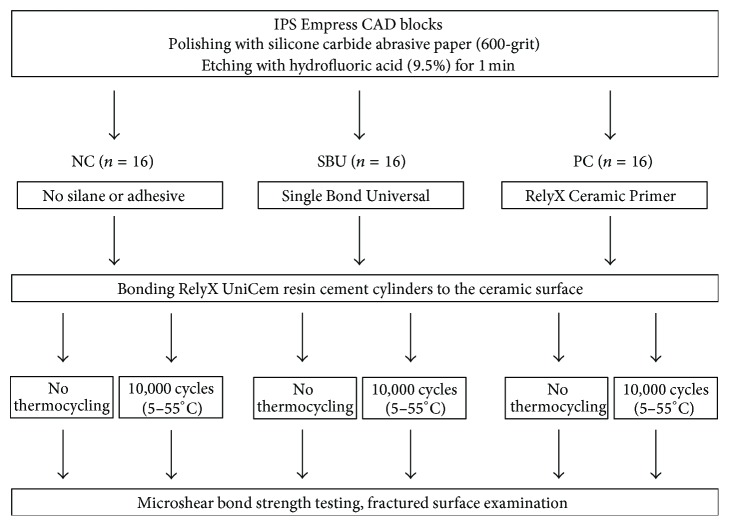
Experimental procedure. NC: negative control; SBU: Single Bond Universal; PC: positive control.

The materials used in this study are shown in Table 1, and the experimental procedure is schematically explained in Figure 1. Using up to 600-grit silicon carbide sandpaper (Rotopol-V, Struers, Ballerup, Denmark), 48 leucite-reinforced glass-ceramic blocks (IPS Empress CAD, Ivoclar Vivadent, Schaan, Liechtenstein) with 14 × 14 mm^2^ surface were polished under running water. The ceramic blocks were acid-etched with 9.5% hydrofluoric acid (Porcelain Etchant, Bisco, Schaumburg, IL, USA) for 1 min, rinsed with water, and then cleaned ultrasonically in isopropyl alcohol for 3 min. The specimens were randomly divided into three groups of 16 samples each. These groups were then subjected to one of the following surface treatment methods:

  NC: negative control (NC); no additional treatment;
  SBU: Single Bond Universal (SBU) adhesive (3M ESPE, St. Paul, MN, USA) was applied for 20 s and the sample was then air-dried for 10 s;
  PC: positive control (PC); RelyX Ceramic Primer (3M ESPE), which consists of silane, was applied for 20 s and the sample was then air-dried for 10 s.

A self-adhesive resin cement (RelyX Unicem Self-Adhesive Universal Resin Cement, 3M ESPE) was mixed and polyethylene tubes (Tygon R-3603 tubing, Saint-Gobain Co., Courbevoie, France) were filled and placed on the specimens. The tubes were light-cured for 20 s each from all four directions at 800 mW/cm^2^ (Elipar Free Light 2, 3M ESPE). After the tubes were removed, a resin cement cylinder of 0.8 mm in diameter and 1 mm in height remained on the ceramic specimen surfaces.

### 2.2. Microshear Bond Strength Measurements

From the total 16 specimens in each group, eight were subjected to *μ*SBS testing after 24 h storage in distilled water at 37°C, while the remaining specimens were subjected to thermocycling for 10,000 cycles at 5 and 55°C, with 25 s dwell time before testing. Using a universal testing machine (LF Plus, Lloyd Instruments, Fareham, UK) at a crosshead speed of 0.5 mm/min, shear force was applied until failure through a stainless steel orthodontic wire of 0.2 mm in diameter, which was positioned as close as possible to the ceramic/resin bond interface.

The *μ*SBS values were analyzed using one-way analysis of variance, and the Tukey honest significant difference (HSD) was used for post hoc testing of any difference between the various surface treatment groups. The effect of thermocycling on each surface treatment group was analyzed using a paired *t*-test. (SPSS software version 21, IBM, New York City, NY, USA) at an *α* level of 0.05.

### 2.3. Examination of Fractured Surfaces

Following *μ*SBS testing, the fractured specimen surfaces were studied under a stereomicroscope (SZ4045, Olympus Optical Co. Ltd., Tokyo, Japan) to determine the mode of failure at 40x magnification, and the precise failure mode was determined. If the fracture occurred within the ceramic, it was categorized as cohesive failure, and if the fracture occurred within both the ceramic and the resin cement, it was deemed a mixed failure. The fractured specimens were also examined with a scanning electron microscope (SEM) at 200x magnification (S-4700 FESEM, Hitachi, Tokyo, Japan).

## 3. Results

The mean and standard deviation of the *μ*SBS (MPa) value for each group are shown in Table 2. Before and after thermocycling (*P* < 0.05), the highest *μ*SBS was exhibited by the PC group specimens, followed by the SBU and NC groups. However, the SBU group exhibited a significant reduction in *μ*SBS after thermocycling (*P* < 0.05), while the other two groups were unaffected. After thermocycling, the PC group exhibited the highest *μ*SBS compared to the other two groups (*P* < 0.05), and the *μ*SBS of the SBU group did not differ significantly from the NC group (*P* > 0.05).

**Figure 2:**
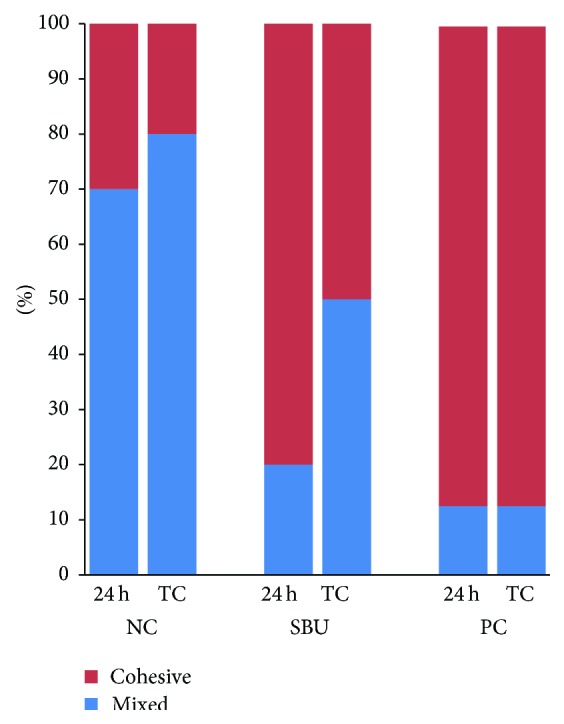
Failure mode distribution after 24 h and 10,000 thermocycles. NC: negative control; SBU: Single Bond Universal; PC: positive control.

**Figure 3:**
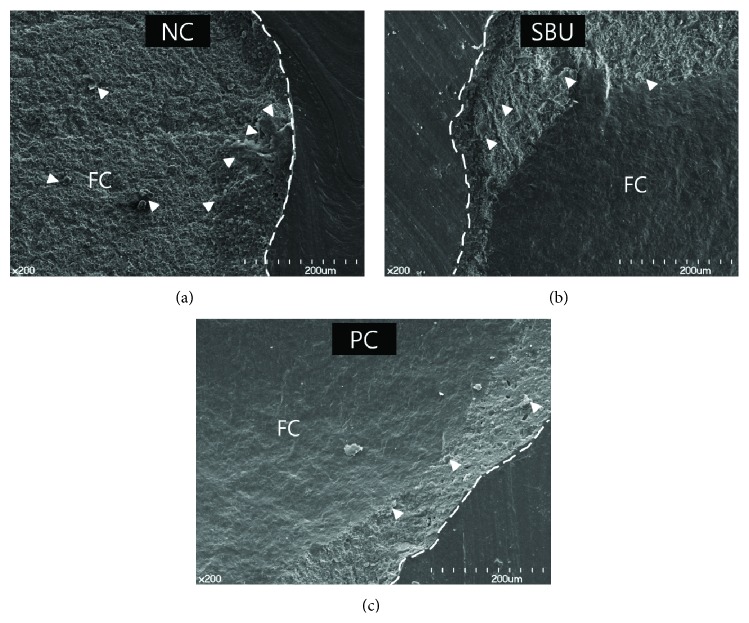
Representative SEM images of fractured ceramic specimens. (a) Facture (dashed lines) occurred within the cemented area in the negative control (NC) group. ((b) and (c)) Fracture occurred (dashed lines) beyond the initial cemented surface in Single Bond Universal (SBU) and pure-saline positive control (PC) groups. The arrowheads show the resin cement remnants on the fractured ceramic (FC). Magnification: 200x.

The distributions of the failure modes for each group are shown in Figure 2. It can be seen that the silane PC group predominantly fractured because of cohesive failure within the ceramic both before and after thermocycling, while the NC group exhibited predominantly mixed failure both before and after thermocycling. However, for the SBU group, thermocycling resulted in an increase in mixed failure and a reduction in cohesive failure within the ceramic. Representative SEM images are shown in Figure 3. More resin remnants are seen in the NC group specimen, while larger and deeper cohesive ceramic fractures are seen in the SBU and PC group specimens.

## 4. Discussion

This study evaluated the *μ*SBS between a self-adhesive resin cement and leucite-reinforced glass-ceramics before and after thermocycling, according to the following different surface treatment methods: no additional treatment (NC group), universal adhesive application (SBU group), and pure-saline application (PC group).

The highest *μ*SBS performance of the self-adhesive resin cement, RelyX Unicem, to the leucite-reinforced glass-ceramic was measured for the PC group, for which pure silane was applied on the ceramic specimen surfaces. This is consistent with the findings of many previous studies, which recommend silane application to increase the bonding between silica-based ceramics and resin composite [12–14, 17, 18]. This performance is expected, as silane's organofunctional terminal groups bond with resin and its hydroxyl groups bond with silica [18, 19].

However, although SBU also contains saline, the *μ*SBS of the SBU group was significantly lower than that of the PC group. In addition, while the SBU *μ*SBS was markedly higher than that of the NC group before thermocycling, the *μ*SBS value decreased significantly after thermocycling to the NC group value. This indicates that while SBU contains silane it is not as effective as pure silane in improving the bond strength between RelyX Unicem and silica-based ceramics. This finding is consistent with the results of Kalavacharla et al. [16], who reported that a silane pretreatment step significantly improved the bond strength when lithium-disilicate was bonded using SBU. This implies that SBU alone is not as effective as pure saline in improving the ceramic-resin bond. The ineffectiveness of the silane in SBU may be due to the fact that various components such as acidic methacryloyloxydecyl dihydrogen phosphate (MDP) and bisphenol A-glycidyl methacrylate (BisGMA) are mixed in a single bottle with silane [18, 20]. As SBU is in an acidic condition due to the acidic monomer, MDP, the silanol groups in silane may undergo premature self-condensation reactions [18]. In addition, BisGMA may prevent the reaction of silane with the hydroxyl group of the ceramic surface containing silica [20].

In the SBU group, a significant reduction in *μ*SBS occurred after thermocycling, lowering the *μ*SBS value to that of the NC group. After thermocycling, the effect of the SBU is lost, and only the micromechanical retention effect remains in place [15]. Since the initial *μ*SBS before thermocycling was significantly higher than that of the NC group, it may be inferred that the increased bond strength of the SBU group after 24 h storage is attributed to the increased wettability and flow in the irregularly etched ceramic surface, rather than true chemical bonding. The observed reduction in the *μ*SBS of the SBU group after thermocycling was consistent with the fracture mode distribution. The favorable cohesive failure within the ceramic decreased after thermocycling, while the mixed failure increased.

The results also show that although the NC group with no additional surface treatment exhibited the lowest *μ*SBS of approximately 20 MPa this value is clinically acceptable. In fact, 10–13 MPa has been suggested as the minimum clinically acceptable *μ*SBS value for bonded restorations [21]. Moreover, no significant reduction in the *μ*SBS of the NC group occurred after thermocycling. The measured 20 MPa *μ*SBS is most likely due to the micromechanical retention achieved by the partial dissolution of the silica-based ceramic surface after etching with hydrofluoric acid [22, 23]. However, when compared with other groups, the NC group exhibited a greater mixed failure distribution with larger resin cement remnants and less ceramic fracture.

Within the limitations of this study, when self-adhesive resin cement was applied to silica-based ceramics, the *μ*SBS values exhibited by specimens with applied SBU or no surface treatment were significantly lower than those exhibited by specimens undergoing separate saline application. While the use of SBU improved the *μ*SBS value, it was not as effective as a pure-saline treatment, and the *μ*SBS of these specimens decreased significantly to that of the untreated group after thermocycling. However, although the *μ*SBS of the untreated group was the lowest measured value, it was clinically acceptable even after thermocycling.

## 5. Conclusions

The *μ*SBS of Rely X Unicem U200, a self-adhesive resin cement, to hydrofluoric acid-etched glass-ceramics was significantly improved by the additional application of SBU or silane. Thermocycling significantly reduced the *μ*SBS of SBU treated group while it did not affect the *μ*SBS of untreated group and the saline treated group.

## Figures and Tables

**Table 1:** Materials used in this study.

Materials (Lot number)	Composition	Manufacturer
IPS Empress CAD (R04751)	Silicon dioxide, aluminium oxide, potassium oxide, sodium oxide, other oxides, pigments (leucite-reinforced glass-ceramic)	Ivoclar Vivadent, Schaan, Liechtenstein

Porcelain Etchant (9.5%) (120006991)	Hydrofluoric acid, polysulfonic acid	Bisco Inc., Schaumburg, IL, USA

Single Bond Universal (539321)	MDP, Bis-GMA, HEMA, decamethylene DAM, ethanol, water, silane treated silica, 2-propenoic acid, -methyl-, reaction products with 1,10-decanediol and phosphorous oxide, copolymer of acrylic and itaconic acid, dimethylaminobenzoate(-4), camphorquinone, (dimethylamino)ethyl methacrylate, methyl ethyl ketone	3M ESPE, St. Paul, MN, USA

RelyX Ceramic Primer (N526043)	Ethyl alcohol, water, methacryloxypropyl-trimethoxysilane	3M ESPE, St. Paul, MN, USA

RelyX Unicem U200 (548681)	*Base*: methacrylate monomers containing acid groups, methacrylate monomers, silanated fillers, initiator components, stabilizer *Catalyst*: methacrylate monomer, alkaline fillers, silanated fillers, initiator components	3M ESPE, St. Paul, MN, USA

**Table 2:** Mean and standard deviation (SD) of microshear bond strength (in MPa).

Group	Water storage (24 hours)	Thermocycling (10,000 cycles)
NC	20.18 (2.01)^c^	20.68 (4.60)^b^
SBU	27.33 (2.81)^b^	22.49 (4.11)^b*∗*^
PC	30.50 (3.40)^a^	28.77 (3.52)^a^

Within the same column, values with different superscript lower case letters are statistically significantly different (Tukey HSD, *P* < 0.05).

*∗* indicates a significant reduction in bond strength for each group after 10,000 thermocycles (paired *t*-test, where *P* < 0.05).

NC: negative control; SBU: Single Bond Universal; PC: positive control.
